# Ultralight Ceramic Fiber Aerogel for High-Temperature Thermal Superinsulation

**DOI:** 10.3390/nano13081305

**Published:** 2023-04-07

**Authors:** Fengqi Liu, Chenbo He, Yonggang Jiang, Junzong Feng, Liangjun Li, Guihua Tang, Jian Feng

**Affiliations:** 1 Science and Technology on Advanced Ceramic Fibers and Composites Laboratory, College of Aerospace Science and Technology, National University of Defense Technology, Changsha 410073, China; liufengqi13@nudt.edu.cn (F.L.); fengj@nudt.edu.cn (J.F.); 2 MOE Key Laboratory of Thermo-Fluid Science and Engineering, School of Energy and Power Engineering, Xi’an Jiaotong University, Xi’an 710049, China

**Keywords:** fiber aerogel, high-temperature superinsulation, structural design, numerical simulation, ultralight, elastic

## Abstract

Emerging fiber aerogels with excellent mechanical properties are considered as promising thermal insulation materials. However, their applications in extreme environments are hindered by unsatisfactory high-temperature thermal insulation properties resulting from severely increased radiative heat transfer. Here, numerical simulations are innovatively employed for structural design of fiber aerogels, demonstrating that adding SiC opacifiers to directionally arranged ZrO_2_ fiber aerogels (SZFAs) can substantially reduce high-temperature thermal conductivity. As expected, SZFAs obtained by directional freeze-drying technique demonstrate far superior high-temperature thermal insulation performance over existing ZrO_2_-based fiber aerogels, with a thermal conductivity of only 0.0663 W·m^−1^·K^−1^ at 1000 °C. Furthermore, SZFAs also exhibit excellent comprehensive properties, including ultralow density (6.24–37.25 mg·cm^−3^), superior elasticity (500 compression cycles at 60% strain) and outstanding heat resistance (up to 1200 °C). The birth of SZFAs provides theoretical guidance and simple construction methods for the fabrication of fiber aerogels with excellent high-temperature thermal insulation properties used for extreme conditions.

## 1. Introduction

Insulation materials for extreme environments, especially for high-speed aircraft thermal protection systems, are subjected to high heat flux and violent vibration, and therefore are required to deliver low density, outstanding insulation properties and reliable mechanical stability [1]. Conventional ceramic aerogels stand out from the crowd with their low thermal conductivity and excellent temperature resistance [2]. However, the inherent brittleness due to the point contact between granular microstructures makes them susceptible to structural collapse under severe impact [3]. The fiber/whisker reinforced aerogel composites exhibit enhanced mechanical properties, but this not only brings a significant increase in density, but also makes it tough to achieve good cladding for complex and precise structures due to the lack of flexibility [4,5]. In addition, the rigid ceramic aerogel composites can hardly be applied in structures with variable dimensions and will suffer from slagging under severe vibrations, causing trouble for structural assembly [6]. Therefore, flexible ceramic aerogels are urgently desired for thermal insulation of complex deformable structures.

The inherent brittleness of ceramics can be substantially improved by the design of the basic structural unit. In recent years, a wide variety of elastic ceramic aerogels have been developed by employing flexible one-dimensional (1D) fibrous building blocks, including SiC nanowires [7,8,9], BN nanoribbons [10,11], oxide nanofibers (SiO_2_ [12,13], ZrO_2_ [14], Al_2_O_3_ [15,16]), etc. Thanks to the lap and entanglement between the continuous fibrous structures, the ceramic aerogels exhibit excellent compressive durability performance [17,18]. Further, through the modulation of pore structure as well as fiber morphology, fiber aerogels have achieved breakthroughs in bending and tensile properties [19,20]. In addition, substantial efforts have been devoted to suppressing the slip and growth of ceramic crystals at high temperatures, such as introducing polycrystalline structures [21], doping foreign elements [16], cladding carbon layers [22], etc., which have led to a significant increase in the thermostability of aerogels (up to 1500 °C). However, as insulation materials for applications under extreme conditions, in addition to mechanical properties and temperature resistance, high-temperature insulation properties are also important evaluation factors that need to be considered.

It is well known that the extremely high porosity of lightweight fiber aerogels can significantly reduce solid heat transfer, resulting in an ultra-low thermal conductivity comparable to that of air at room temperature [12,23]. However, due to the massive macropores and extremely low density, which are not conducive to the suppression of gas heat transfer and dominant radiative heat transfer at high temperatures, the thermal conductivity of fiber aerogels increases significantly with elevated temperatures [22,24], which greatly limits their further applications in extreme environments. After compounding with nanoporous SiO_2_ aerogels, although the room temperature thermal conductivity of aerogel composites can be further reduced to 0.024 W·m^−1^·K^−1^ thanks to the effective suppression of gas heat transfer [14], it does not function at high temperatures because of the infrared transparency of SiO_2_ aerogels. The hollow structured fibers can effectively reduce the solid heat transfer, but the infrared shading properties of the aerogels are not fundamentally improved [25,26]. Hence, the preparation of fiber aerogels with superior high-temperature thermal insulation properties remains a great challenge.

Commonly, the addition of infrared opacifiers with high specific extinction coefficients, such as SiC [27], TiO_2_ [28,29] and carbon [24,30], is an effective approach to suppress the high-temperature radiation heat transfer of conventional aerogels, but this method has not been widely applied to fiber aerogels. Moreover, fiber orientation also has a crucial effect on high-temperature thermal conductivity of conventional fiber-reinforced aerogel composites [31,32,33], but this has not been verified in fiber aerogels. To the best of our knowledge, there are few reports focusing on the modulation of high-temperature thermal insulation properties of fiber aerogels so far, especially the simultaneous regulation of composition, fiber arrangement and shading agents. Among these few reports, Guo et al. [24] introduced carbon into the fiber to improve the infrared shading performance of the aerogel, but the carbon was prone to be oxidized in air at high temperatures. Zhang et al. [34] reduced the high-temperature thermal conductivity by growing rigid TiO_2_ hollow spheres and nanowires on the fibers, but this was at the expense of the flexibility of the aerogels. Therefore, as emerging aerogels, more in-depth theoretical and experimental studies are still needed to enhance the heat insulation performance at high temperatures and thus improve their reliability in practical applications.

Here, focusing on the modulation of high-temperature thermal insulation performance of fiber aerogels, we first optimize the composition and structure of the aerogel based on numerical simulation. The results show that the fiber aerogels with low high-temperature thermal conductivity can be obtained by doping appropriate content of SiC shading agent in the directionally arranged ZrO_2_ fiber aerogels (SZFAs). Guided by this and combined with the unidirectional freezing technique, ultralight fiber aerogels with desired structures were then prepared by using ZrO_2_ fibers and SiC particles as the basic building blocks. Benefiting from the multiscale structure and the excellent thermal stability of ZrO_2_ fibers, the SZFAs can be repeatedly compressed to 60% strain for 500 times and remain structurally stable over a wide temperature range (−196–1100 °C). More importantly, thanks to the synergistic effect of directionally arranged ZrO_2_ fibers and SiC opacifiers, the thermal conductivity of SZFAs at 1000 °C is as low as 0.0663 W·m^−1^·K^−1^, which is the lowest value ever reported for ZrO_2_-based fiber aerogels. This work provides a new vision for the preparation of fiber aerogels for thermal insulation in extreme environments.

## 2. Materials and Methods

### 2.1. Materials

ZrO_2_ fibers with a diameter of about 1 μm were purchased from Shandong Dongheng New Material Co., Ltd. SiC particles (99.9%) with a diameter of about 1–3 μm were from Shanghai Chaowei Nano Co., Ltd. Polyethylene oxide (PEO, Mv~5000000), tetraethyl orthosilicate (TEOS, 98%), aluminum chloride (99.9%) and boric acid (99.5%) were all from Macklin Co., Ltd. (Shanghai, China). Deionized water was self-made from the laboratory ultrapure water machine.

### 2.2. Preparation of ZFAs and SZFAs

A self-made unidirectional freezing platform was obtained by placing the bottom end of copper rods in liquid nitrogen. Aluminoborosilicate (ABS) sols were prepared by mixing TEOS, aluminum chloride and boric acid in water in the Al/Si/B molar ratio of 2:5:1 as in [12]. A certain amount of ZrO_2_, SiC particles, 2 g ABS sol and 0.5 g PEO were added to 100 g deionized water and a homogeneous dispersion was obtained after stirring at 5000 rpm·min^−1^ for 30 min. The dispersion was solidified on the unidirectional freezing platform and then vacuum dried for 72 h to obtain pre-SZFAs, and SZFAs were produced after calcination at 900 °C for 1 h. By simply enlarging the mold, the size of the sample can reach 15 × 15 × 2 cm. The mass ratio of fiber to water was defined as fiber content, and pure ZrO_2_ fiber aerogels (ZFAs) with fiber content of 0.3%, 0.5%, 1%, 1.5% and 2% were defined as ZFA-0.3, ZFA-0.5, ZFA-1.0, ZFA-1.5 and ZFA-2.0, respectively. The mass ratio of SiC particles to fiber was defined as SiC content. Taking the sample with 2% fiber content as an example, samples with SiC contents of 10%, 20%, 30, 40% and 50% are defined as S10ZFA-2.0, S20ZFA-2.0, S30ZFA-2.0, S40ZFA-2.0 and S50ZFA-2.0, respectively. The heat-treated samples were obtained by heating in a muffle furnace to the target temperature (1000–1300 °C) and holding for 1 h (heating rate 10 °C·min^−1^).

### 2.3. Characterization

SEM images and EDS mappings were taken by a ZEISS scanning electron microscope (Sigma 300, Oberkochen, Germany). Tecnai G2F20 transmission electron microscope (FEI, Hillsboro, OR, USA) was employed to take TEM micrographs. XRD patterns were tested by a PANalytical diffractometer (Empyren, Almelo, The Netherlands). A universal tester (FL4204GL, Fule Test Technology) was applied to test mechanical properties at a moving rate of 1 mm·min^−1^. Temperature change on the sample surface was tested by an M200A infrared camera (InfiRay, Hefei, China). Room temperature thermal conductivity was measured by the anisotropic module of Hotdisk (TPS2000s, Uppsala, Switzerland) in the direction perpendicular to the fiber direction (in the radial direction). The high-temperature thermal conductivity of the samples was also obtained by the high-temperature test probe of Hotdisk. The UV–VIS-NIR spectrophotometer (Lambda 750s, Waltham, MA, USA) was employed to measure the spectral transmittance of the ZrO_2_ fiber aerogels in the range of 0.5–1.4 μm. A quartz cuvette with a width of 1 mm was used during measurements, and an empty quartz cuvette was used as a reference. The spectral transmittance of the ZrO_2_ fiber aerogels was measured by the FTIR spectrometer (INVENIO R, Oberschleibheim, Germany) in the range of 1.4–25 μm. The infrared pressing pellet of the samples was prepared by using KBr powder as the diluents during measurements.

### 2.4. Extinction Efficiency of a Single Fiber

The extinction efficiency of a single fiber is a basis to determine the radiative characteristics of the fiber aerogel. Fibers with high aspect ratio can be reasonably assumed as infinitely long cylinders, and the extinction efficiency (*Q*_*ext*,*f*_) of fiber (ZrO_2_, Al_2_O_3_, SiO_2_) can then be obtained from applying Mie-scattering theory to the cylinder [35,36,37]:

(1) Qext,Iϕ=2χRe⁡b0I+2∑n=1∞bnIQext,IIϕ=2χRe⁡a0II+2∑n=1∞anIIQext,fϕ=12Qext,Iϕ+Qext,IIϕ

where *ϕ* is the incident angle between the fiber axis and incident radiation, *Re* denotes the real part of a complex quantity, *χ* = *πd*/*k* is the size parameter at the wavelength *k*, and *d* is the diameter of the fiber. *b*_0,I_, *b*_*n*,I_, *a*_0,II_ and *a*_*n*,II_ are the Mie coefficients. A detailed description of Mie-scattering theory was given in [35]. The Matlab functions by Schäfer are used in this study for calculating *Q*_*ext*,*f*_ of a single fiber [37].

### 2.5. Simulation of Heat Conduction in Directionally Arranged ZrO_2_ Fibers Network

In this work, the preprocessing (including generation of representative volume element (RVE) and application of temperature boundary condition), finite element analysis and postprocessing (including generation of heat flux field) are conducted in the commercial finite element package ABAQUS to simulate heat transfer (combined effect of solid and gas heat transfer) in fiber networks [38]. The mesh-size independence was tested and convergence of results was obtained.

### 2.6. The Radiative Thermal Conductivity of SZFAs

The spectral transmittance of ZrO_2_ fiber aerogels based on UV-VIS-NIR spectrophotometer and FTIR measurements in the spectral range of 0.5–25 μm. According to the Lambert–Bell law [39], the spectral extinction coefficient *β*_*λ*,*ae*_ of ZrO_2_ fiber aerogels can be obtained from the measured spectrum transmittance *τ*_*k*_, as:

(2) $$\beta_{\lambda ,\mathrm{ae}}=\beta_{\lambda ,\mathrm{ae}}^{\prime }\rho =\frac{-\ln\left(\tau_{\lambda }\right)}{L}$$

where *β*^′^_*λ*,*ae*_ is the specific spectral extinction coefficient, *ρ* is the density of samples, *τ_λ_* is the transmittance of samples, *L* is the thickness of samples and *_λ_* is the wavelength. The effective thickness of samples in the infrared pressing pellets *L_pellet_* could be determined as [39]:

(3) $$L_{\mathrm{pellet}}=\frac{M_{\mathrm{pellet}}\omega }{\rho A_{\mathrm{cross}}}$$

where *M_pellet_* is the weight of the pellet, *ω* is the mass proportion of samples in the pellet and *A_cross_* is the cross section area of the pellet. Assuming that opacifiers can be regarded as spheres, they are distributed uniformly in the ZrO_2_ fibers network and the independent scattering condition is satisfied. The complex refractive index *m* = *n* + *ik* of the SiC has two variables [40], the refractive index *n* and the absorptive index *k*. Then the extinction efficiency *Q*_*ext*,*o*_ for a single opacifier can be well described by Mie-scattering theory applied to particle [35,37]:

(4) Qext,om,χ=2χ2∑n=1∞2n+1Re⁡an+bn

where *d* is the diameter of the opacifier, and *a_n_* and *b_n_* are the Mie coefficients. *χ* is the size parameter at the wavelength *k.* A detailed description of Mie-scattering theory was given in [35]. The Matlab functions by Schäfer are used for calculating *Q*_*ext*,*o*_ of a single opacifier [37]. In this situation, the spectral extinction coefficient *β*_*k*,*o*_ of SiC opacifiers is given by [41]:

(5) $$\beta_{\lambda ,o}=\frac{3Q_{\mathrm{ext},o}f_{v}}{2d}$$

where *f_v_* is the volume fraction of SiC opacifiers. The conversion relationship between volume fraction and mass fraction can be expressed as [42]:

(6) $$\mathrm{wt}=\frac{f_{v}\rho_{\mathrm{sic}}}{\rho_{\mathrm{ae}}}$$

where *ρ_SiC_* is the density of SiC doped-ZrO_2_ fiber aerogel, and *ρ*_*ae*_ is the density of SiC opacifier. Further, when the doping mass fractions of SiC opacifiers are 10%, 20%, 30%, 40% and 50%, the corresponding volume fractions are 0.12%, 0.27%, 0.45%, 0.71% and 0.98% calculated by Equation (6). By averaging the spectral extinction coefficients of the blackbody radiation distribution, the temperature dependence of the average extinction coefficient of functional doping can be obtained [41]:

(7) $$\frac{1}{\beta \left(T\right)}=\int_{0}^{\infty }\frac{1}{\beta_{\lambda }}\frac{\partial E_{b\lambda }}{\partial E_{b}}d\lambda$$

where *E_b_* is defined as the blackbody emissive power, *E_bk_* is is identified as the spectral blackbody emissive power and *T* is the temperature. Thus, the total extinction coefficient of ZrO_2_ fiber aerogel composites doped with SiC opacifiers, *β_total_* (*T*) can be expressed as:

(8) $$\beta_{\mathrm{total}}\left(T\right)=\beta_{o}\left(T\right)+\left(1\;-f_{v}\right)\beta_{\mathrm{ae}}\left(T\right)$$

where *β_f_* (*T*) is the extinction coefficient of SiC particles and *β_ae_* (*T*) is the extinction coefficient of the ZrO_2_ fiber aerogels matrix. The radiative thermal conductivity (*k_r_*) of SiC opacifiers-doped ZrO_2_ fiber aerogels is [41]:

(9) $$k_{r}=\frac{16}{3\beta_{\mathrm{total}}\left(T\right)}\sigma T^{3}=\frac{16}{3\rho e(T)}\sigma T^{3}$$

where *σ* is the Stefan–Boltzmann constant, *e* is the specific extinction coefficient of the aerogel and *ρ* is the material density. The effective thermal conductivity (*k_e_*) of SiC opacifiers-doped ZrO_2_ fiber aerogels is the summation of the conductive thermal conductivity (*k_c_*) (combined effect of solid thermal conductivity and gas thermal conductivity) and the radiative thermal conductivity (*k_r_*), as [43]:

(10) $$k_{e}=k_{c}+k_{r}$$

where the conductive thermal conductivity (*k_c_*) can be calculated by the Maxwell model as [44]:

(11) $$k_{c}=\left[1+\frac{3\left(\alpha -1\right)f_{v}}{\left(\alpha +2\right)-\left(\alpha -1\right)f_{v}}\right]k_{\mathrm{ae}}$$

where *α* = *k_p_*/*k_ae_*, *k_o_* is the thermal conductivity of SiC opacifiers [45] and *k_ae_* is the thermal conductivity of ZrO_2_ fiber aerogel matrix.

## 3. Results

### 3.1. Structural Design of Fiber Aerogels Based on Numerical Simulation

**Figure 1:**
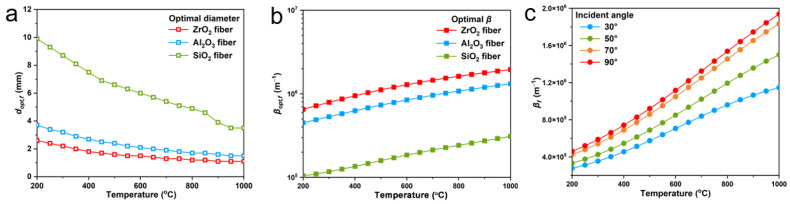
The radiative properties of three fibers. (**a**) Optimal cylinder diameter of various fibers. (**b**) Optimal temperature-dependent extinction coefficient of fibers based on the optimal diameter. (**c**) Temperature-dependent extinction coefficient of ZrO_2_ fibers against angle between fiber axis and incident radiation.

To obtain fiber aerogels with low thermal conductivity at high temperature, the fiber composition needs to be screened prior to the structural design. Common oxide ceramic fibers including SiO_2_, ZrO_2_ and Al_2_O_3_ fibers were compared. The extinction efficiency of the three types of fibers were calculated by Equation (1). The complex refractive indexes of ZrO_2_, Al_2_O_3_ and SiO_2_ are picked by [46,47,48] in the spectral range of 0.5 to 25 μm. The maximum temperature-dependent mean extinction coefficient of fibers is calculated based on the optimal fiber diameter at that temperature [33]. The optimal diameters of fibers at various temperatures are presented in Figure 1a, and the maximum temperature-dependent mean extinction coefficient of fibers based on the optimal diameter is shown in Figure 1b. The results show that among the three fibers, ZrO_2_ fibers have the best extinction performance in the full temperature range of 200–1000 °C, and the optimal diameter of ZrO_2_ fibers gradually decreases with increasing temperature from 2.6 μm at 200 °C to 1.1 μm at 1000 °C. Therefore, considering the high-temperature application scenario of aerogels, ZrO_2_ fibers with an average diameter of 1 μm were chosen as the basic unit for fabricating the fiber aerogels.

**Figure 2:**
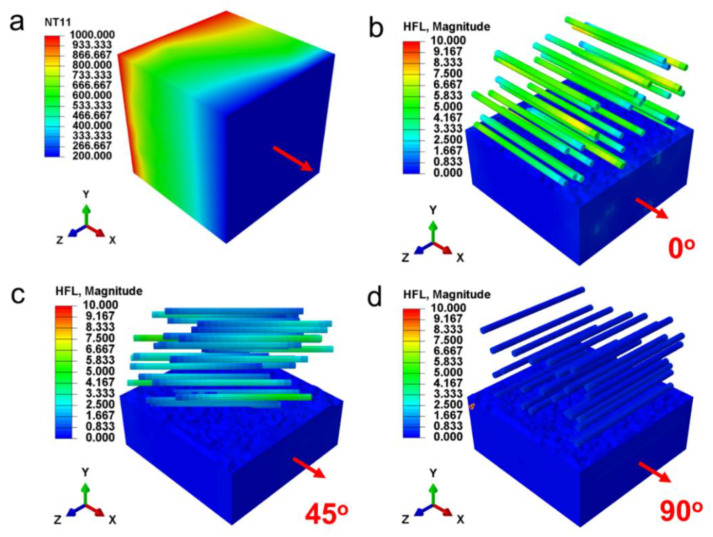
The heat conduction properties of ZrO_2_ fibers network. (**a**) Temperature gradient vector of RVE. The angles between fiber axis and temperature gradient vector are (**b**) 0°, (**c**) 45° and (**d**) 90°.

The fiber has significant anisotropy and its orientation direction also has a pronounced effect on both conductive thermal conductivity (*k_c_*) and radiative thermal conductivity (*k_r_*). Hence, the impacts of fiber alignment direction on shading performance of fiber aerogel were first investigated. Mie-scattering theory is applied to evaluate the radiation characteristics of ZrO_2_ fibers (1 μm diameter) at various angles between fiber axis and incident radiation (Figure 1c). The results reveal that ZrO_2_ fibers possess the maximum temperature-dependent mean extinction coefficient when the fiber axis is perpendicular to the incident radiation, i.e., the incident angle of 90°. The relationship between fiber alignment direction and heat conduction (comprehensive effect of solid and gas conduction) was also discussed. To simulate the heat conduction of ZrO_2_ fibers with fiber axis perpendicular or parallel to temperature gradient vector by using RVE based finite element method (FEM) [49], we firstly generate the periodic RVEs of the directionally arranged ZrO_2_ fiber network with the aspect ratio of 20 and fiber volume fraction of 5% by the random sequential absorption (RSA) algorithm [50]. The steady state heat transfer of a RVE with the prescribed temperature boundary condition is considered, and the temperature gradient vector is along the x-axis (Figure 2a). The heat flux fields of the directionally arranged ZrO_2_ fiber network by the FEM simulation is shown in Figure 2b–d. The results demonstrate that the heat flux fields of RVE with fiber axis perpendicular to temperature gradient vector (90°) achieve the minimum value. According to the Fourier’s law [50], the *k_c_* of directionally arranged ZrO_2_ fiber network is the lowest when the fiber axis is parallel to the temperature gradient vector. Therefore, the fibers arranged perpendicular to the heat flux can obtain both lowest *k_c_* and *k_r_*, achieving the best thermal insulation property of ZrO_2_ fiber aerogels.

**Figure 3:**
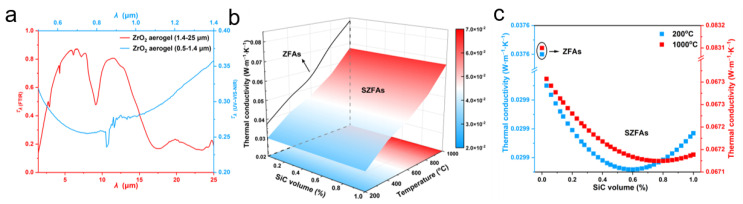
The radiative properties of ZFAs and SZFAs. (**a**) Spectral transmittance of the ZFAs. (**b**) Thermal conductivity of ZFAs and SZFAs. (**c**) Thermal conductivity of ZFAs and SZFAs at 200 and 1000 °C.

According to Equation (9), *k_r_* is proportional to *T*^3^ and increases dramatically with temperature, causing radiation become the dominant heat transfer mode at high temperatures. Therefore, to suppress the thermal radiation inside the ZrO_2_ fiber aerogels, it is necessary to dope opacifier particles to further enhance the aerogel shading performance. Among several common ceramics (ZrO_2_, TiO_2_ and SiC), SiC exhibits superior extinction properties and is therefore chosen as an opacifier in this work [33]. However, doping SiC opacifiers in ZrO_2_ fiber aerogels will also inevitably increase the solid heat transfer. Thus, there exists an optimal SiC amount that can well balance the radiative and solid heat transfer, achieving a minimum value of thermal conductivity for aerogels. Firstly, the spectral transmittance of ZrO_2_ fiber aerogels (ZFAs) is measured based on UV-VIS-NIR spectrophotometer and FTIR measurements in the spectral range of 0.5–25 μm (Figure 3a). Then, the temperature-dependent radiative thermal conductivity of SiC opacifiers doped-ZrO_2_ fiber aerogels (SZFAs) is calculated for a wide range of doping volume fractions (0–1%) based on the spectral transmittance of ZFAs, and the optimal doping volume fraction of SiC opacifiers is obtained. The results show that the effective thermal conductivity of SZFAs decreases significantly after being opacified with SiC particles (Figure 3b,c), indicating that the radiative transmission could be well restrained by SiC opacifiers. And the optimal doping volume fraction of SiC opacifiers gradually increases from 0.59% at 200 °C (corresponding to mass fractions between 30 and 40%) to 0.78% at 1000 °C (corresponding to mass fractions between 40 and 50%), which provides a guide for subsequent material preparation.

### 3.2. Fabrication and Characterization of SZFAs

According to the above structural design, the appropriate content of the shading agent is expected to be incorporated uniformly into the directionally arranged ZrO_2_ fiber network. For conventional fiber-reinforced aerogel composites, the micron-sized opacifier particles in the sol are susceptible to being blocked and intercepted by the high-density fiber mat during the impregnation process, resulting in the inhomogeneous distribution of the opacifier in the composite. Thus, the in situ growth strategy of the opacifier is encouraged for conventional aerogel composites [51]. However, this also brings a more complicated process and a higher cost. The case changes for the freeze-drying method, where the dispersion of fibers and opacifier can be transformed into a homogeneous three-dimensional (3D) porous structure by the simple “one-pot” and casting-sublimation process. In addition, the fiber alignment direction can be manipulated by controlling the ice crystal growth pattern. Hence, in this regard, freeze-drying is an ideal method to fulfill the above structural design principles.

**Figure 4:**
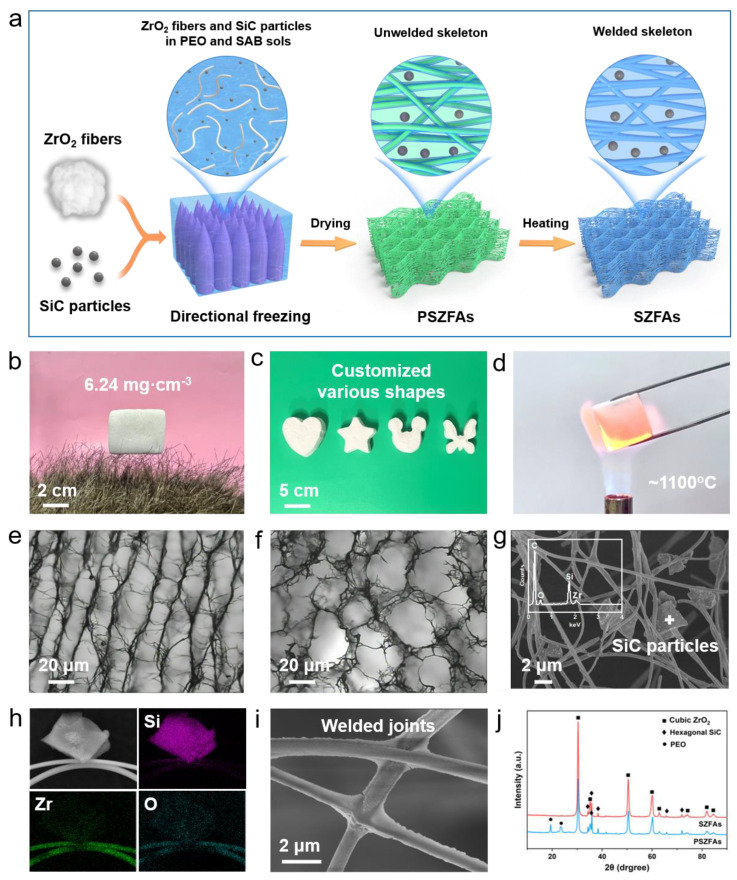
(**a**) Schematic diagram of the preparation process of SZFAs. (**b**) Macroscopic photograph of SZFAs standing on artificial hairs. (**c**) Customized SZFAs with different shapes. (**d**) SZFAs heated by 1100 °C flame without damage. Microscopic photographs of SZFAs in (**e**) radial and (**f**) axial directions. (**g**) SEM image of SZFAs and EDS pattern of SiC particles. (**h**) EDS mapping images of Si, Zr and O elements in SZFAs. (**i**) SEM image of inter-fiber welded joints. (**j**) XRD patterns of PSZFAs and SZFAs.

Figure 4a illustrates the preparation procedure of SiC-doped ZrO_2_ fiber aerogels (SZFAs). ZrO_2_ nanofibers and SiC particles are homogeneously dispersed in a polyethylene oxide (PEO) dispersion containing aluminoborosilicate (SAB), and pre-SZFAs (PSZFAs) with unwelded skeletons are obtained via directional freezing and vacuum drying processes. After calcination at 900 °C, SZFAs with stable honeycomb structures were successfully prepared. PEO acts as a dispersant and thickener to avoid settling of fibers and particles during freezing, while SAB serves as a bonding agent to weld fiber-fiber and fiber-shading agent together during calcination, thus obtaining a robust 3D porous skeleton. Compared with conventional granular aerogels, SZFAs can maintain an intact porous structure at very low densities due to the self-lap and winding effect between continuous fibers. By varying the content of fibers in the dispersion, the density of SZFAs can be arbitrarily adjusted from 6.24 to 37.24 mg·cm^−3^. As shown in Figure 4b, the ultralight SZFAs can be stably supported by several artificial hairs without falling off, demonstrating the ultralight properties. Besides, SZFAs can be tailored to any desired shape by simply changing the mold shape (Figure 4c), which lays the foundation for its application in complex structures. Notably, the excellent thermal stability of ZrO_2_ fibers also enables SZFAs to maintain structural integrity even under the heating of a butane torch (~1000 °C) (Figure 4d).

During the freeze etching process, the directionally growing ice crystals can exclude the fibers and SiC particles that occupy their growth path, forcing the fibers to align along the ice crystal growth direction in the ice crystal gap. As expected, the SZFAs exhibit a multiscale anisotropic structure (Figure 4e,f). In the radial direction (perpendicular to the ice crystal growth direction), the ordered fiber bundles are aligned in parallel with an average spacing of about 20 μm, which provides the structural basis for their excellent thermal insulation performance in this direction. In the axial direction (parallel to the ice crystal growth direction), the fibers surround the holes created by the ice crystal etching, forming a honeycomb structure. As observed in the enlarged SEM image (Figure 4g), SiC particles with the size of 1–3 μm are uniformly distributed in the fiber network without any obvious aggregation, and the composition of SZFAs is also confirmed by the distributions of Si, Zr and O elements in the EDS mapping image (Figure 4h). The cross-lap joints between the fibers are firmly welded by SAB (Figure 4i), which avoids inter-fiber slippage and structural collapse under stress. The XRD pattern showed that the peaks attributed to PEO disappeared after calcination due to oxidative decomposition, but the diffraction peaks attributed to SZFAs did not change significantly because of the excellent temperature resistance of ZrO_2_ fibers and SiC particles (Figure 4j).

### 3.3. The Elasticity and Temperature Resistance of SZFAs

**Figure 5:**
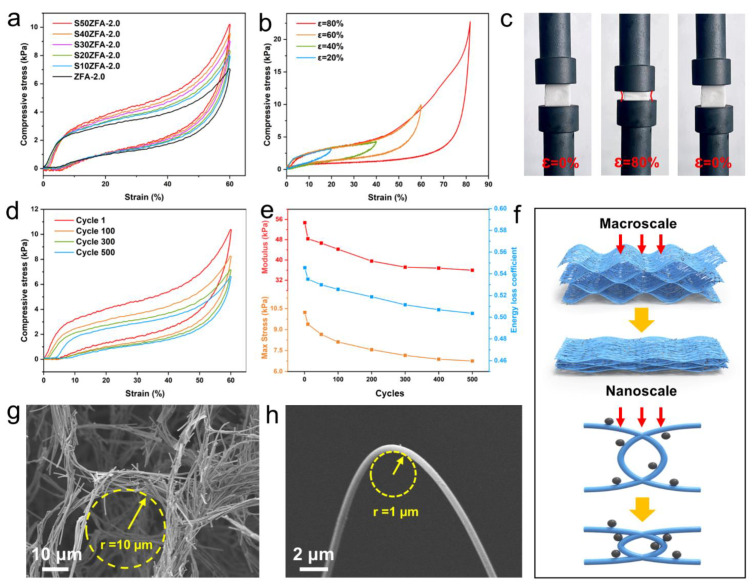
(**a**) Stress–strain curves (60% strain) for ZFA-2.0 and SZFAs with different SiC contents. (**b**) Stress–strain curves of S50ZFA-2.0 at different strains. (**c**) Photograph of the compression process of S50ZFA-2.0 (80% strain). (**d**) Compression cycle curves of S50ZFA-2.0 (60% strain, 500 cycles). (**e**) Curves of elastic modulus, maximum stress, and energy loss coefficient with increasing compression cycles. (**f**) Schematic of the elastic deformation mechanism. Curvature radius of (**g**) a single honeycomb structure and (**h**) a single fiber.

In contrast to the brittle particle ceramic aerogels, SZFAs exhibit excellent mechanical properties as well as compression cycling stability. Figure 5a compares the compressive properties of SZFA-2.0 with different SiC contents. It can be observed from the stress-strain (ε) curves that all the samples experience three typical stages during the compression process, including the elastic deformation stage (ε < 5%), the plateau stage (5% < ε < 50%) and the densification stage (50% < ε). Since micron-sized SiC particles can perform a supporting role as the honeycomb pore walls approach each other, the compressive stress increases gradually with the increase of SiC content in the plateau and densification stages. At 60% strain, the maximum compressive strength increased from 7.06 kPa for ZFA-2.0 to 10.22 kPa for S50ZFA-2.0. Further, the mechanical properties of S50ZFA-2.0 at strains ranging from 20% to 80% were investigated (Figure 5b). S50ZFA-2.0 can recover to its initial shape even at 80% strain (Figure 5c), accompanied by a maximum compressive strength of 18.81 kPa, which means that it can withstand more than 4000 times its own weight. To demonstrate the mechanical durability of S50ZFA-2.0, it was compressed for 500 cycles at 60% strain, exhibiting a plastic deformation of only 3.91% (Figure 5d). As observed in Figure 5e, 79.4% maximum compressive strength and 80.9% elastic modulus can still be retained after 100 compression cycles, and the strength does not deteriorate significantly, even after 500 compression cycles, exhibiting 66.1% retention of compressive strength and 65.7% retention of modulus. The modest contraction of the hysteresis curve also implies a slight reduction in the energy loss coefficient (from 0.546 to 0.504 after 500 cycles), emphasizing the long-term structural stability of S50ZFA-2.0.

For conventional aerogels composed of ceramic nanoparticles, the fragile neck connection fails to act as a buffer when subjected to external stresses [52], triggering permanent structural damage rather than elastic recovery. In addition, previous reports have also suggested that the disordered random fiber networks can only withstand small deformation [53,54]. Therefore, it can be speculated that the excellent dynamic resilience of SZFAs is mainly attributed to the synergistic effect of the macroscopic honeycomb pore structure as well as the flexible fibers. At the macroscopic scale (Figure 5f), the ordered honeycomb pore structure contracts under compression, while the cell walls formed by interwoven fiber bundles with a curvature radius of about 10 μm can effectively transfer the load and dissipate the stress by bending and twisting, exerting a buffering effect. Meanwhile, the ultrahigh porosity of SZFAs (>99.9%) means that the pore wall structures of the honeycomb will not contact and squeeze with each other even under high strain, thus avoiding lateral expansion and exhibiting a negative Poisson’s ratio (Figure 5c). At the nanoscale, the flexible ZrO_2_ fibers are able to avoid fracture under large bending deformation with a curvature radius of 1 μm, so that the fibers arranged perpendicular to the load direction can resist more severe deformation by conforming to the parallel load direction under the force. Besides, the SiC particles can also support the approaching fibers at high strain, leading to an increase in compressive strength (Figure 5f). Benefiting from the cooperation of multiscale structures, no serious stress concentration and structural damage occur inside the 3D network, so the aerogel can be reverted to its initial state after removing the load.

**Figure 6:**
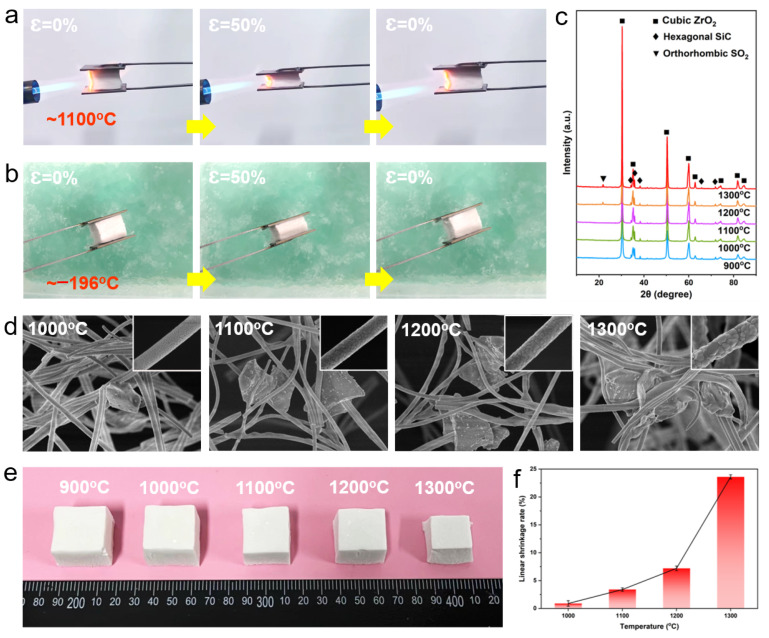
In situ compression experiments in (**a**) butane torch (~1100 °C) and (**b**) liquid nitrogen (~−196 °C). (**c**) XRD patterns of S50ZFA-2.0 after heat treatment at different temperatures. (**d**) SEM photographs and enlarged views of fibers (inset) of S50ZFA-2.0 after heat treatment at different temperatures. (**e**) Macroscopic photographs and (**f**) linear shrinkage rate of S50ZFA-2.0 after heat treatment at various temperatures.

To demonstrate the wide working temperature range of SZFAs, the samples were subjected to in situ compression tests in a butane torch flame (~1100 °C) and liquid nitrogen (~−196 °C). As shown in Figure 6a,b, under extreme high/low temperature environments, SZAs could still remain elastic and recover to their original state from 50% strain without any visible cracks. The crystal evolution process of S50ZFA-2.0 at high temperature was also investigated by XRD (Figure 6c). Owing to the excellent thermal stability of cubic ZrO_2_ and the protective effect of the SiO_2_ layer on the SiC particles, the diffraction peaks due to ZrO_2_ and SiC in S50ZFA-2.0 become sharper as the temperature increases from 900 to 1300 °C, implying an increase in crystallinity and the growth of the grains, but without crystalline phase transformation. Note that a faint diffraction peak appears at 22° for temperatures higher than 1200 °C, which is associated with the transformation of the SiO_2_ protective layer from the amorphous phase to the orthorhombic crystalline phase at high temperatures [14]. The SEM images of S50ZFA-2.0 at different temperatures show that a noticeable change is not observed in the SiC particle morphology with increasing temperature (Figure 6d), but the ZrO_2_ fibers undergo significant grain growth as well as inter-fiber fusion and adhesion above 1200 °C, which will lead to an increase in fiber brittleness. Figure 6e,f shows the macroscopic photographs and liner shrinkage rate curves of S50ZFA-2.0 after heat treatment at different temperatures. In line with the SEM results, S50ZFA-2.0 exhibits only slight shrinkage at temperatures below 1200 °C (0.9% and 3.4% at 1000 and 1100 °C, respectively). With further increase in temperature, the sample shrinkage increased violently from 7.2% at 1200 °C to 23.6% at 1300 °C due to the sintering of the fibers, so the upper limiting service temperature of SZAs were considered to be 1200 °C.

### 3.4. Thermal Insulation Properties of SZFAs

In addition to excellent mechanical properties and temperature resistance, outstanding high-temperature thermal insulation performance is also a crucial prerequisite for the application of SZFAs in extreme environments. However, for nanofiber aerogels, the low density of the 3D network structure and the low specific extinction coefficient of the fibers have a limited effect on the suppression of radiative heat transfer, which generally leads to a high thermal conductivity at elevated temperatures (>0.1 W·m^−1^·K^−1^ at 1000 °C). According to the design of the material composition and structure in Section 3.1, adding an appropriate amount of SiC shading agent to the directionally arranged ZrO_2_ nanofiber aerogel is expected to obtain fiber aerogels with low high-temperature thermal conductivity. Based on this, both pure ZrO_2_ nanofiber aerogels (ZFAs) and SiC-doped ZrO_2_ nanofiber aerogels (SZFAs) were prepared for comparison.

**Figure 7:**
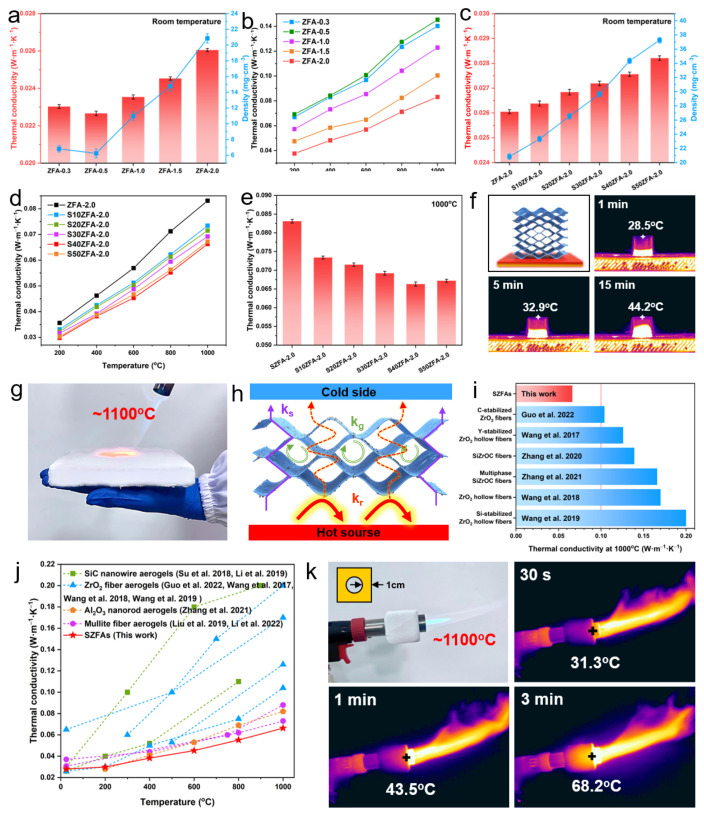
(**a**) Density and room temperature thermal conductivity as well as (**b**) high-temperature thermal conductivity of ZFAs with different fiber contents. (**c**) Density and room temperature thermal conductivity as well as (**d**,**e**) high-temperature thermal conductivity of SZFAs with different SiC contents. (**f**) Hot table heating experiment of S50ZFA-2.0 (300 °C). (**g**) Superior thermal insulation performance of S40ZFA-2.0 for protecting a hand (heated for 1 min) from damage under 1100 °C flame. (**h**) Schematic of high-temperature superinsulation mechanism of S50ZFA-2.0. (**i**) Comparison of high-temperature thermal conductivity of SZFAs with the reported ZrO_2_-based fiber aerogels at 1000 °C [24,57,58,59,60,61]. (**j**) Comparison of high-temperature thermal conductivity between SZFAs and reported fiber aerogels [9,15,16,21,24,57,60,61,62]. (**k**) Demonstration experiment of high-temperature thermal superinsulation performance of SZFAs.

For porous materials with a pore size below 1 mm, convective heat transfer can be neglected [55], and the total effective thermal conductivity of a material is the summation of gas thermal conductivity (*k_g_*), solid thermal conductivity (*k_s_*) and radiation thermal conductivity (*k_r_*) [4,56]. *k_r_* accounts for a small proportion at relatively low temperature [34], so the total thermal conductivity of aerogels at room temperature is attributed to *k_g_* and *k_s_*. Figure 7a shows the density and room temperature thermal conductivity of ZFAs with different fiber contents. As the fiber content decreases from 2% to 0.5%, the density of the aerogel gradually decreases from 20.85 mg·cm^−3^ for ZFA-2.0 to 6.24 mg·cm^−3^ for ZFA-0.5. However, with further decrease in fiber content, the density of ZFA-0.3 increases slightly (6.81 mg·cm^−3^), which is due to the fact that the extremely low content fiber backbone suffers more severe shrinkage during calcination. Higher density means more transfer paths for *k_s_*, resulting in higher thermal conductivity. Therefore, the thermal conductivity follows the same trend as the density, reaching a minimum value of 0.0226 W·m^−1^·K^−1^ at 0.5% fiber content. At high temperatures (Figure 7b), the situation is reversed and the thermal conductivity of ZFAs decreases with increasing density, from 0.1452 W·m^−1^·K^−1^ for ZFA-0.5 to 0.0831 W·m^−1^·K^−1^ for ZFA-2.0 at 1000 °C, which can be explained by the fact that *k_r_* mainly contributes to the thermal conductivity at high temperatures [4,56]. According to Equation (9), for ZFAs, the increase in density brings about a decrease in *k_r_*, resulting in an overall decrease in thermal conductivity.

ZFA-2.0 exhibits a lower high-temperature thermal conductivity, so the doping of shading agent is further carried out on the basis of this fiber density. It can be found from Figure 7c that both the density and room temperature thermal conductivity of SZFA-2.0 monotonically increase with the addition of SiC particles. The increase in *k_s_* is not significant due to there being less of a lap between uniformly dispersed SiC particles. Therefore, although the density of the sample increases substantially from 23.33 mg·cm^−3^ for S10ZFA-2.0 to 37.25 mg·cm^−3^ for S50ZFA-2.0, the thermal conductivity merely goes up slightly from 0.0261 to 0.0283 W·m^−1^·K^−1^. As expected, after doping SiC into ZFAs, the high-temperature thermal conductivity of SZFAs decreases remarkably, and this superiority becomes more significant with increasing temperature. At 1000 °C, the thermal conductivity of the sample declined significantly, by over 20% from 0.0831 W·m^−1^·K^−1^ for ZFA-2.0 to 0.0663 W·m^−1^·K^−1^ for S40ZFA-2.0 as the SiC content varied from 0 to 40%. However, when the SiC content continued to increase, the thermal conductivity of S50ZFA-2.0 conversely grew to 0.0672 W·m^−1^·K^−1^, which results from the competitive effect between radiative and solid-state heat transfer. According to Equation (9), the addition of SiC significantly boosts the specific extinction coefficient of SZFAs, thus substantially suppressing the dominant *k_r_* and reducing the total thermal conductivity. Nevertheless, the decrease in *k_r_* by excessive SiC can no longer compensate for the increase in *k_s_*, so that the thermal conductivity shows an increasing trend for SiC contents higher than 40%. Thus, S40ZFA-2.0 with 2% fiber content and 40% SiC content exhibits the lowest high-temperature thermal conductivity, which coincides with the numerical results in Section 3.1.

Hot plate heating experiments were performed to visually demonstrate the excellent thermal insulation properties of S40ZFA-2.0. The sample with fibers perpendicular to the direction of heat flow was placed on a 300 °C hot plate, and its upper surface temperature slowly rose from 28.5 °C at 1 min to 32.9 °C at 5 min, and only increased to 44.2 °C after 15 min of heating. S40ZFA-2.0 features even more significant thermal insulation superiority at extreme temperatures. Surprisingly, thanks to the ultra-low high-temperature thermal conductivity, the 2-cm-thick S40ZFA-2.0 could effectively block the heat from the 1100 °C flame, and the hand was not hurt even after heating for 1 min (Video S1). The powerful high-temperature insulation performance of SZFAs is mainly attributed to the synergistic effect of the directionally aligned ZrO_2_ nanofibers and the SiC opacifiers (Figure 7e): (i) In terms of material composition, ZrO_2_ fibers with a diameter of 1 μm have a higher specific extinction coefficient than SiO_2_ and Al_2_O_3_ fibers. Besides, the addition of opacifiers further improves the shading performance of the aerogel. (ii) In terms of material structure, the fibers arranged perpendicular to the heat flow direction maximizes the suppression of heat transfer. (iii) The ultra-high porosity and suitable density of SZFAs enable the *k_g_* and *k_s_* at a low level. Benefiting from the comprehensive material design, the high-temperature thermal insulation performance of SZFAs is much better than that of existing ZrO_2_-based fiber aerogels, and the previously reported minimum value of thermal conductivity for ZrO_2_-based fiber aerogels at 1000 °C is still 56.9% higher than that of SZFAs (Figure 7i) [24,57,58,59,60,61]. As shown in Figure 7j, the high-temperature thermal insulation performance of SZFAs also presents significant advantages over other ceramic fiber aerogels, including SiC nanowire aerogels [9,62], ZrO_2_ fiber aerogels [24,57,60,61], Al_2_O_3_ nanorod aerogels [16] and mullite fiber aerogels [15,21]. To simulate actual application scenarios in extreme environments, S40ZFA-2.0 was customized into a 1 cm thick thermal protection jacket for a butane gun nozzle (~1100 °C). After 3 min of heating, the maximum surface temperature of S40ZFA-2.0 only increased from 31.3 to 68.2 °C. This means that a 1 cm thick sample can even cause a temperature decay of over 1000 °C, demonstrating reliable thermal insulation performance at extreme temperatures.

## 4. Conclusions

In summary, by coupling theoretical design and material preparation, we successfully prepared ultralight honeycomb structured fiber aerogels with ultra-low high-temperature thermal conductivity by using ZrO_2_ fibers and SiC particles. With ultra-low density (6.24–37.25 mg·cm^−3^), excellent compressive elasticity (500 cycles at 60% strain), exceptional heat resistance (up to 1200 °C), and superinsulation properties (0.0283 W·m^−1^·K^−1^ at 25 °C 0.0663 W·m^−1^·K^−1^ at 1000 °C), SZFAs are expected to be applied as insulation materials in extreme environments, especially for the protection of structures with complex shapes and variable dimensions. Moreover, the structure that facilitates high-temperature insulation and the preparation method for SZFAs are discussed in detail, which is a versatile technique and can be applicable to other types of fiber aerogels. The present work provides ample opportunities for the development of high-temperature superinsulating fiber aerogels.

## Data Availability

Data is contained within the article.

## Supplementary Materials

The following supporting information can be downloaded at: https://www.mdpi.com/article/10.3390/nano13081305/s1, Video S1: Superior thermal insulation performance of S40ZFA-2.0 for protecting a hand (heated for 1 min) from damage under 1100 °C flame.
